# Central and peripheral mechanisms of narcotic antitussives: codeine-sensitive and -resistant coughs

**DOI:** 10.1186/1745-9974-3-8

**Published:** 2007-07-09

**Authors:** Kazuo Takahama, Tetsuya Shirasaki

**Affiliations:** 1 Department of Environmental and Molecular Health Sciences, Graduate School of Pharmaceutical Sciences, Kumamoto University, 5-1 Oe-Honmachi, Kumamoto 862-0973, Japan

## Abstract

Narcotic antitussives such as codeine reveal the antitussive effect primarily via the μ-opioid receptor in the central nervous system (CNS). The κ-opioid receptor also seems to contribute partly to the production of the antitussive effect of the drugs. There is controversy as to whether δ-receptors are involved in promoting an antitussive effect. Peripheral opioid receptors seem to have certain limited roles. Although narcotic antitussives are the most potent antitussives at present, certain types of coughs, such as chronic cough, are particularly difficult to suppress even with codeine. In guinea pigs, coughs elicited by mechanical stimulation of the bifurcation of the trachea were not able to be suppressed by codeine. In gupigs with sub-acute bronchitis caused by SO_2 _gas exposure, coughing is difficult to inhibit with centrally acting antitussives such as codeine. Some studies suggest that neurokinins are involved in the development of codeine-resistant coughs. However, evidence supporting this claim is still insufficient. It is very important to characterize opiate-resistant coughs in experimental animals, and to determine which experimentally induced coughs correspond to which types of cough in humans. In this review, we describe the mechanisms of antitussive effects of narcotic antitussives, addressing codeine-sensitive and -resistant coughs, and including our own results.

## Introduction

Cough causes via the activation of cough reflex arc consisted of the airway vagal afferent nerves, cough center in the medulla and the efferent nerves. Inhibiting it at any site of the arc can be expected to cause antitussive effect. However, the mechanisms of cough generation, its modulation and antitussive effect of centrally and peripherally acting antitussives are still largely unclear. Of the many available narcotic and non-narcotic antitussives, the most effective are the narcotic antitussives, which are of limited use due to their inherent undesirable side effects, particularly their narcotic side effects. Even for this codeine, it has recently been pointed that it is not effective as estimated from the experimental results in guinea pigs [1]. Also, chronic coughs are often resistant to treatment with codeine. Thus, there is a need for new types of antitussives that can suppress chronic coughs. It is unclear why some coughs, such as chronic cough, are resistant even to treatment with potent antitussives such as codeine, although it is known that coughing is a neural reflex. In this review, we discuss the mechanisms of the effects of narcotic antitussives on coughing using experimental animals, and further, the resistance of coughs to narcotic antitussives, describing our recent findings regarding codeine-sensitive and -insensitive coughs in guinea pigs.

## Opioid receptor subtypes and antitussive effects

The antitussive mechanisms of narcotic antitussives are not fully understood. The available evidence clearly indicates that narcotic antitussives act on opioid receptors [2-4]. Binding studies concerning guinea-pig and human opioid receptors demonstrated that codeine and dihydrocodeine, gold standard narcotic antitussives, were more selective to the μ-opioid receptor than other κ- or δ-opioid receptors [3,5]. K_i _value of [^3^H]codeine (3.7 × 10^-7 ^M) for replacement of [^3^H]-D-Ala^2^, MePhe^4^, Gly-ol^5^] enkephalin (DAMGO), a μ-selective ligand, in guinea pigs [3] was close to the K_D _value of 5.6 × 10^-7 ^M for the saturable binding of [^3^H]codeine in the lower brain stem of guinea-pigs [6]. μ-Selective morphine has much more potent antitussive activity in cat [7]. κ-Agonists also have antitussive activity. Therefore, both μ- and κ-opioid receptors have been considered as candidates for being the receptors which contribute to antitussive activity.

Further, pharmacological studies carried out by using rats, mice and μ_1_-opioid receptor deficient mice suggested that μ_2_- rather than μ_1_-subtype of the μ-opioid receptor contributes to the antitussive activity of opioids [8,9]. Unfortunately, there is argument against these results in mice and rats, because it has been unable to reliably obtain a cough-like behavior in mice and rats. In addition, Ohi *et al*. [10] recently found that the motor patterns of rats and guinea pigs during cough-producing stimuli were significantly different. In rats, two different types of behavior were observed and one of them did not conform to the classic definition of a cough. Codeine suppressed both behaviors. For these reasons, it has been addressed that rats and mice are not viable as models of cough.

This issue seems to cast its shadow over the conflicting results about the role of δ-opioid receptors in producing the cough inhibiting effect of narcotic antitussives. Kamei *et al *[11] demonstrated that [D-Pen^2,5^]enkephalin (DPDPE), a selective δ-agonist, did not have an antitussive effect, but rather inhibited the antitussive effects of DAMGO and K-50488H, a selective κ opioid receptor agonist found in rats. But, δ-antagonists such as naltrindole and naltriben reduced the number of capsaicin-induced coughs in mice and rats [12,13]. Mu- and κ-opioid receptor antagonists did not antagonize the δ-antagonist-induced antitussive activities. Conversely, Kotzer *et al*. [5] showed that the highly selective δ-agonist SB 227122 inhibited the cough-reflex induced by citric acid in guinea-pigs. The antitussive effect of SB227122 was antagonized by the δ-antagonist SB 244525. This δ-antagonist itself did not have an antitussive effect. Kotzer *et al*. have also reported that naltrindole binds to human μ- and κ-opioid receptors at significant levels. Further studies are required to confirm whether the controversy presented above comes from differences in the species of experimental animals used and/or differences in the pharmacological properties of each δ receptor agonist and antagonist used.

Apart from the above, we have recently found evidence for another possible mechanism of the antitussive effects of δ-antagonists. In a patch clamp study using single brain neurons, naltrindole and naltriben both inhibited the currents caused by activation of G-protein-coupled inwardly rectifying K^+ ^(GIRK) channel [14]. GIRK channels couple to the 5-HT_1A _receptor, and contribute to a negative feedback mechanism of 5-HT release. Dextromethorphan, which is a representative non-narcotic antitussive and has an inhibitory effect on GIRK channel activated currents [15], antagonized the 5-HT-induced hyperpolarization and depolarized the membrane potential, generating action potentials in dorsal raphe neurons. Thus, inhibition of this channel may increase the 5-HT level in the CNS. In human volunteers, infusion of 5-HT or its precursor reduced cough responses to a chloride-deficient solution [16]. In contrast, reduction of 5-HT levels has been found to inhibit the antitussive effects of narcotic and non-narcotic antitussives [17]. Stimulation of raphe nuclei depresses discharges in inspiratory motoneurons [18,19]. The 5-HT_1A _receptor agonist inhibited cough responses, although it stimulated cough response at high doses [20]. 5-HT_2_/5-HT_1 _receptor antagonists inhibited any morphine-induced antitussive effect in humans [21]. In addition, DMGO increased 5-HT efflux in dorsal raphe nucleus [22]. Taken together, the above findings suggest that antitussive effects of δ-antagonists are at least partly due to the inhibition of GIRK channel currents [23].

Next, we will discuss the site of antitussive action of opioids in the CNS. Results of *in vivo *experiments suggest that centrally acting antitussives primarily act on the brainstem cough center. Recently, Gestreau *et al*. [24] reported that fictive cough selectively increased Fos-like immunoreactivity (FLI) in the interstitial and ventrolateral subdivision of the nucleus tractus solitarius (NTS), the reticular formation (the medial part of the lateral tegmental field, and the internal division of the lateral reticular nucleus), the ambigual complex (the nucleus retroambiguus, the para-ambigual region, and the retrofacial nucleus), and the medial parabrachial nucleus in cat. In all the nuclei, codeine significantly reduced the increase in FLI. Further, laryngeal afferent stimulation enhanced FLI in periaqueductal gray matter (PAG) and dorsal raphe nucleus in cat [25].

μ-Opioid receptors are expressed intensely or moderately in the ambiguus nucleus, NTS, dorsal vagal nerve nucleus, medial parabrachial nucleus, PAG and raphe nuclei [26-29]. In these regions, κ-opioid receptors are also expressed with similar or less potent density. δ-Opioid receptors are generally less abundant in the brainstem, but the pneumotaxic center, including the nucleus parabrachialis, contains a very high density of δ-binding site. In the NTS and ambiguus nucleus, it is expressed weakly. Here, caudal NTS and its neighboring ventromedial region has been considered as a strong candidate for being the cough center, because this region primarily receives sensory input from the lower airway [30,31] and its stimulation causes cough-like response [32,33]. The NTS is more heavily labeled by the μ-ligand than by the κ-ligand in guinea pigs and cats [29,34]. Further, the μ_2 _sites have been found to be associated with respiratory depressant effects of opioids, whereas the μ_1 _sites have been found to be associated with the analgesic effects of opioids in mouse brain [35]. Microinjection of codeine into the NTS inhibited a fictive cough reflex in guinea pigs [36]. μ-Opioid receptor agonist presynaptically inhibited excitatory postsynaptic currents in the NTS [37]. Kappa- and δ-opioid receptor agonists also inhibited excitatory postsynaptic potentials in the NTS but they are less effective than μ-opioid receptor agonist [38].

Given these together with the reported affinity of narcotic antitussives for opioid receptors, narcotic antitussives might have a primary site of antitussive effect on μ-opioid receptors in the NTS, although there is a report that the antitussive effects of codeine are not blocked by naloxone in cats [38]. In addition to the NTS, the raphe nuclei may be a candidate for being the site of action of narcotic antitussives, since stimulation of the raphe nuclei depresses the reflex activity caused by stimulation of the superior laryngeal or vagal nerve in respiratory interneurons of the NTS, without affecting respiratory rhythm [39]. This characteristic seems to be in accordance with properties that antitussives are presumed to have.

## Peripheral opioid receptors and antitussive effects

Mu-opioid receptors are located in both the central and peripheral nervous systems. Adcock [40] has written a nice review about the sensory opioid receptor and antitussive activity of narcotic antitussives. Inhalations of nebulized codeine, morphine and a peripherally acting specific μ-opioid receptor agonist produced antitussive effects in guinea pigs [41,42]. Therefore, it is plausible that inhaled opioid antitussives exert their effect by inhibiting tachykinergic transmission of excitatory non-adrenergic non-cholinergic (eNANC) nerves via a blockade of μ-opioid receptors in the airway, although it is unknown whether opioids affect peripheral opioid receptors when administered via conventional routes.

In addition to the effect on sensory fibers, opioid agonists also appear to inhibit airway cholinergic transmission [43-45]. Opioid-induced inhibition of the cholinergic bronchoconstriction induced by electric field stimulation (EFS) in guinea-pig is caused partly by an inhibitory action on the eNANC nerve, and partly by a direct effect on cholinergic transmission [44]. The inhibitory effect of μ-opioid ligands on EFS-induced cholinergic contraction of the airway's smooth muscle was also found in human preparation. This effect is presumably caused by inhibiting the acetylcholine release from the postganglionic parasympathetic nerve fibers [45]. Here, controversial opinions exist as to whether the airway contraction induces a cough response or not. However, it has been known that coughing in patients with cough variant asthma [46] is inhibited by bronchodilators such as adrenergic β_2 _stimulants [47]. This fact seems to indicate that the kind of cough such as that found in cough variant asthma may be caused by smooth muscle contraction in the airway.

Postganglionic parasympathetic nerve fibers in the airway arise from the paratracheal ganglia (PTG). Their excitability is controlled by the preganglionic neurons via central vagal reflex. In addition, they can be modulated by a peripheral reflex mechanism because the collateral branches of neurokinin-containing C-fibers project to the PTG neurons [48] and enhance cholinergic transmission in the PTG, probably via neurokinin releases [49]. Further, we have recently found that bradykinin inhibits the M-type K^+ ^current in the acutely dissociated PTG neurons of rats, causing depolarization and action potential generation [50]. In addition, bradykinin potentiated nicotinic ACh currents in PTG neurons [51]. Thus, the PTG are thought to be not only a relay neuron of the parasympathetic nerve, but also integrative sites for the neuromodulation of normal airway function and important for pathogenesis in airway inflammation. Interestingly, ophiopogonin-D, an active constituent of bakumondo-to, a Chinese herbal medicine, hyperpolarized the membrane potential via activation of the K^+ ^current, reducing the cell excitability of PTG neurons [52]. Bakumondo-to is found to be effective for treating clinically chronic coughs [53,54], and to inhibit codeine-registrant coughs as that expressed in the experimental model described above [55,56]. Therefore, we speculate that the excitability of PTG neurons may contribute to pathological condition, including some kinds of chronic cough. In this context, we examined the effects of codeine in dissociated PTG neurons. However, codeine did not induce any currents in PTG neurons, and had no effect on high-voltage-activated (HVA) Ca^2+^, bradykinin- induced or nicotine-induced currents in the neurons. Bradykinin-induced potentiation of nicotinic currents in the neurons was also not affected by codeine (unpublished data).

To summarize this section, μ-opioid receptors locate in the airway vagal sensory neurons and, at least inhaled opioids, inhibit both eNANC nerve activity and cholinergic contraction of smooth muscles through acting on μ-opioid receptors. The PTG neurons seem to be a possible target for peripherally acting antitussives. However, opioids have no effect on the PTG neurons.

## Codeine-sensitive and -resistant coughs

The larynx is the most sensitive site for elicitation of the cough reflex by mechanical stimulation, followed by tracheal bifurcation and the lower half of the trachea, in that order [57]. We have recently found that coughs elicited by mechanical stimulation of the tracheal bifurcation were relatively resistant to suppression by codeine in guinea pigs, whereas mechanically induced coughs in the trachea close to the larynx were effectively inhibited by codeine [58].

Sensory receptors in airway vagal afferents have been classified into 5 groups, which include rapidly adapting receptors (RARs), Aδ-nociceptors and bronchial C-fiber receptors. These 3 receptors listed above appear to contribute to cough responses. RARs are myelinated Aδ fibers and have a low threshold for mechanical stimuli, but are resistant to chemical stimuli. Conversely, Aδ-nociceptors and unmyelinated C-fiber receptors have a high threshold for mechanical stimuli, but a low threshold for chemical stimuli such as bradykinin and capsaicin. Recently, a 6^th ^receptor group called "cough receptor" has been identified [59]. Its properties are similar to those of RARs, but they have a slower conduction velocity and did not respond to stretching. In the larynx and upper trachea, "cough receptors" appear to play a primary role in regulation of the cough response [59]. Research by Widdicombe [57] indicated that the larynx and the tracheal bifurcation are abundantly innervated by RARs which presumably include "cough receptors". Conversely, chemoreceptors involved in cough responses are mainly distributed in the lower trachea, particularly around the tracheal bifurcation. Thus, differences in codeine resistivity between areas of the lower airway may be due to differences in the distribution of these various types of sensory fibers.

In guinea pigs, the effects of codeine on mechanically elicited coughs at each lower airway site were strengthened by repeated treatment with large doses of capsaicin [58]. This capsaicin treatment caused degeneration and dysfunction of the C- and Aδ-nociceptors [60-63] and consequently reduced cough generation caused by citric acid and capsaicin, but not coughs caused by nicotine or mechanical stimulation [64]. In addition, angiotensin-converting enzyme inhibitors (ACEIs), which sensitize nociceptive fibers, induced codeine-resistant chronic cough in conscious guinea pigs [65]. Consequently, it has been hypothesized that coughs mediated by nociceptive fibers were resistant to codeine. In our own study, capsaicin administered topically to the tracheal bifurcation caused a cough response that was resistant to codeine, whereas topical application to the larynx side of the trachea did not cause a cough response [66]. In a preliminary histochemical study using guinea pigs, we found that substance P (SP)-like immunoreactivity is lower in the larynx side of the trachea than in the tracheal bifurcation. In addition, the density of SP-immunoreactive nerves has been found to be significantly higher in patients with cough-variant asthma than in normal subjects and patients with classic asthma [67]. The above findings support the hypothesis that coughs mediated by nociceptive fibers may be resistant to codeine treatment.

**Figure 1:**
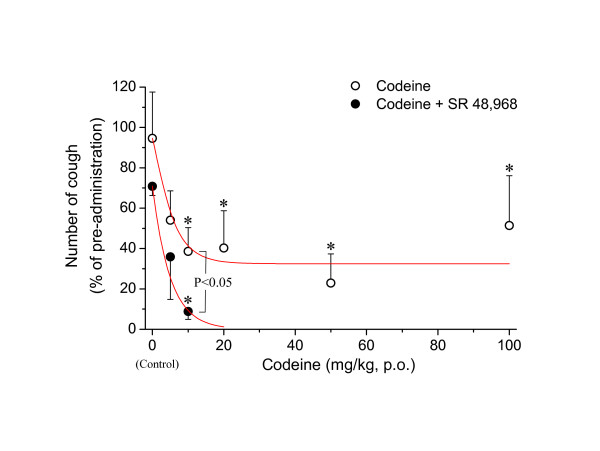
Effect of co-administration of codeine and SR 48,968, a NK_2 _receptor antagonist, on citric acid-induced coughs in conscious guinea-pigs. Conscious guinea-pigs were put into plethysmograph and 10 % citric acid was nebulized for 2 min to elicit a cough response. Cough number was counted for 15 min during and after citric acid stimulation. After more than 4 h, codeine was orally administered at various doses 30 min before the 2^nd ^stimulation. Then, SR 48,968 (1 mg/kg) or vehicle was intravenously administered 5 min before the 2^nd ^stimulation. Results were normalized to the pre-administration cough number. Continuous lines indicate the theoretical fitting of the data with single exponential function. Note that co-administration of codeine (10 mg/kg) and SR 48,968 inhibited cough response almost completely, while the antitussive effect induced by codeine alone reached a plateau at 20 mg/kg (33 % of pre-administration value). Antitussive effects produced by co-administration of codeine (10 mg/kg) and SR 48,968 was significantly more potent than that produced by codeine (10 mg/kg) alone (p < 0.05, n = 4 and 6, respectively). SR 48,968 itself inhibited cough response to about 70 % of the time, but the inhibitory effect was not significantly different from the vehicle group. Co-administration of codeine and SR 48,968 had no effect on mechanically-induced sneezing (data not shown). Each value shows mean ± S.E.M. (n = 3 to 7). * *p *< 0.05, significantly different from the vehicle control.

In a chronic bronchitis model of rats produced by SO_2 _gas exposure, SP content in the trachea was elevated [68]. In a similar model using guinea pigs, codeine did not inhibit the cough responses elicited by mechanical stimulation of the larynx side of the trachea or the tracheal bifurcation. Epithelial shedding was not observed, but neutral endopeptidase (NEP) levels and NEP activity in the trachea and bronchus were significantly lower than those of normal guinea pigs. NEP degrades a variety of peptides, including bradykinin, SP and other tachykinins [69]. At high doses, a NEP inhibitor elicits a cough response in normal guinea pigs [70]. Based on findings that bradykinin and tachykinins are potent inflammatory mediators, and that neurokinins such as SP are released from C-fiber (eNANC nerve) terminals, it has been suggested that coughing induced by inflammatory peptides is resistant to codeine. However, codeine has been found to significantly suppress the cough response induced or enhanced by NEP inhibitors [70]. In addition, opioids peripherally inhibit tachykinergic transmission in the guinea pig bronchus [71-73]. Thus, it appears that NEP inhibition or tachykinin release from peripheral C-fiber terminals is not sufficient to explain mechanisms of induction of codeine-resistant cough. However, in a preliminary study, we found that inhaled neurokinin A caused codeine-resistant cough in guinea pigs. Also, in that study, co-administration of codeine and an antagonist for the neurokinin 2 (NK_2_) receptor almost abolished citric acid-induced coughing in conscious guinea pigs, in spite of the fact that citric acid-induced coughs were hard to completely inhibit even with high doses of codeine, when codeine alone was given (Fig. 1).

In summary, evidence suggests that RAR or "cough receptor"-mediated coughs are sensitive to codeine but coughs triggered by neurokinin-containing nociceptive nerves are resistant to it. In support of this suggestion, there is a finding that the expression of transient receptor potential vanilloid-1 (TRPV-1) is increased in the airway nerves of patients with chronic cough [74]. In addition to TRPV-1, it has recently been reported that acid sensing ion channels (ASICs) were localized in Aδ-fibers of guinea pigs [75]. Therefore, differences in codeine sensitivity to acid-induced coughs may depend on the pH level at the chough induction site. Further studies are needed to determine a final conclusion.

## Codeine resistant coughs and opioid receptors

As described in the above session, evidence suggests that tachykinin-containing vagal afferent fibers contribute to codeine-resistant cough. Such fibers rise from the airway and input the NTS [30,31]. Codeine-resistant coughs are caused by various conditions such as cigarette smoking, infection and inflammation of the airway. Exposure to cigarette smoke augmented the C-fiber input to the NTS [76]. Injection of a NK_1 _receptor antagonist into the NTS had an antitussive effect in animals exposed to cigarette smoke, but not to filtered air [77]. An excitatory action of iontophoretically applied SP on NTS neurons was not inhibited by μ-agonists [78]. These findings seem to suggest that μ-opioid receptors were not expressed in the C fiber such as tachykinin-containing fibers involved in the production of codeine-resistant cough. Furthermore, it has been reported that infection and inflammation of the airway led to the production of neurokinins in nonnociceptive RAR nerve terminals and in their cell bodies in vagal sensory ganglia [79-83]. Sensory neuropeptide release from peripheral and central endings of nonnociceptive afferent nerve seems not to require noxious or nociceptive stimuli but may occur as a result of stimulation of low-threshold mechanosensors [81]. Certainly, codeine only weakly inhibited coughs in animal models of allergic responses [84-86], as well as of chronic bronchitis produced by SO_2 _gas exposure [68]. Therefore, under pathological conditions described above in the airway, changes in phenotype of the vagal nerves and tachykinin release from RAR fibers might facilitate glutamatergic transmission in the nucleus involved in cough reflex, leading to codeine-resistant cough.

As described previously, μ-opioid receptors are the predominant type of opioid receptor in the NTS [87,88]. They exist in both postsynaptic and presynaptic sites in the NTS [38,89]. The μ-agonist activated the GIRK channel current and hyperpolarized the postsynaptic membrane potential in about 60 % of NTS neurons. At the same time, μ-agonists inhibited HVA Ca^2+ ^currents in the nodose ganglia [90], and also the glutamatergic EPSCs in almost all NTS neurons via a presynaptic mechanism, which is much more sensitive to μ-agonist than postsynaptic mechanisms [37,38]. Here, the nodose ganglion is the origin of RAR [91], and glutamate is the principal neurotransmitter in the Aδ-fibers [92-94]. Excision of the nodose ganglion causes marked depletion of μ-opioid receptors in the dorsal and medial regions of the ipsilateral caudal NTS [34]. Electron microscopy has shown that μ-opioid receptors localize in the plasma membrane of the terminals of vagal afferents derived from nodose ganglia [95], but not in nociceptive fibers [96]. Considering these findings comprehensively, it seems possible that μ-receptors in the NTS may not express and/or may not function in neural networks for cough production, under pathological conditions such as infection and inflammation of the airway, although the receptors are involved in codeine-sensitive coughs under healthy conditions without infection and inflammation of the airway.

In opposition to the idea about the relation between neurokinins and codeine-resistant cough, there is an argument based in the fact that neurokinin-containing airway fibers are very few in humans. However, neurokinin-containing vagal airway afferent neurons are only 3% of total neurons, even in guinea-pigs [80]. Comparative study also indicated that the location of SP-immunoreactive fibers in the airway was similar between humans and guinea pigs, although SP-reactive nerves in the smooth muscle layer of the trachea and bronchi were less abundant in rat and cat than in guinea-pig [97]. In addition, as in the cases of animal models, the density of SP-immunoreactive nerves was significantly higher in patients with cough-variant asthma than in normal subjects and patients with classic asthma [67]. Increase in neurokinin content has also been observed in patients with perennial allergic rhinitis [98]. Therefore, it is reasonable to speculate that neurokinin-containing airway afferent nerves play pathophysiological roles in the generation of inflammatory and allergic airway diseases including production of codeine-resistant chronic coughs in humans.

## Conclusion

Based on the above findings, we have developed the following hypothesis. Coughs mediated by mechanical stimulation of RARs or "cough receptors" are attenuated by narcotic antitussives primarily at the NTS level via inhibition of glutamatergic transmission. Presynaptic μ-opioid receptors probably contribute to this inhibition. Conversely, neurokinin release in the NTS from nociceptive C- and Aδ-fibers, and also from RAR fibers under airway inflammation, causes coughs resistant to antitussives including opiates. Recent findings by Mazzone *et al*. [99] support this hypothesis. The available evidence suggests that activation of μ-opioid and inhibition of neurokinin receptors can help in the suppression of some varieties of chronic cough. In a preliminary study, we found that coadministration of codeine and a NK_2 _tachykinin receptor antagonist abolished citric acid-induced coughs in guinea pigs, although codeine alone did not abolish the cough even when administered at very high doses. Further studies are needed to clarify the pharmacology and mechanisms of antitussive effects of opiates, and to elucidate mechanisms of opiate-resistant coughs. Furthermore, it is very important to characterize opiate-resistant coughs in experimental animals, and to determine the extent to which such experimentally induced coughs correspond to the various types of cough in humans.
